# Giant perforating pilomatricoma with osseous metaplasia^☆^

**DOI:** 10.1016/j.abd.2023.05.012

**Published:** 2024-06-08

**Authors:** Vânia Olívia Coelho de Almeida, Ana Carolina Monteiro de Camargo, Meire Soares de Ataíde, Romes José Tristão, Tullio Novaes Silva

**Affiliations:** a Department of Dermatology, Universidade Federal do Triângulo Mineiro, Uberaba, MG, Brazil; b Department of Surgical Pathology, Universidade Federal do Triângulo Mineiro, Uberaba, MG, Brazil

Dear Editor,

A 34-year-old male patient, a microentrepreneur, sought dermatological care due to a tumor measuring approximately 6x5 cm in diameter, with erythematous infiltrated edges, and extrusion of whitish stone-like material in its central portion, on the posterolateral region of the right arm (Fig. 1). The reported evolution was approximately two years and six months. An excisional biopsy was performed with wide margins and histopathology showed an expansive growth neoplasm (Fig. 2), consisting of basaloid cells and “ghost cells” (Fig. 3), affecting the subcutaneous adipose tissue and showing metaplastic osseous metaplasia, dystrophic calcification (Fig. 4), ulceration, and a chronic inflammatory process associated with a gigantocellular reaction of the foreign body type and stromal fibrosis, compatible with perforating pilomatricoma with osseous metaplasia.

**Figure 1:**
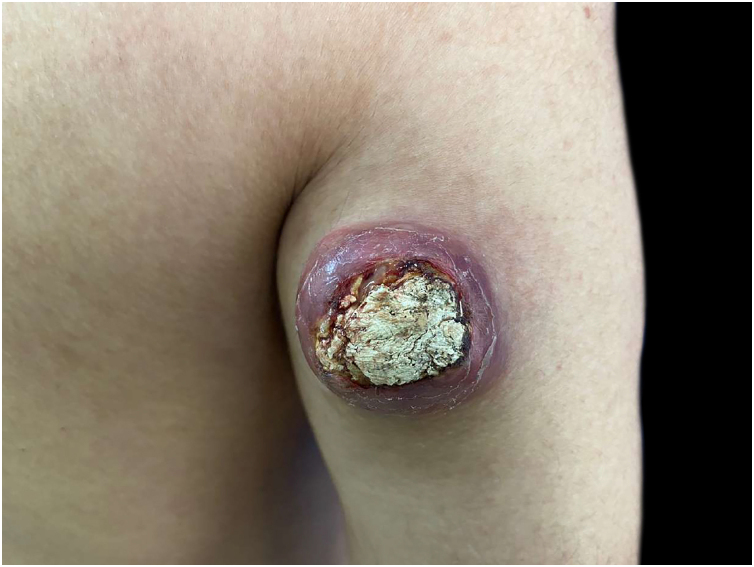
Tumor measuring approximately 6×5 cm in diameter, on the posterolateral region of the right arm.

**Figure 2:**
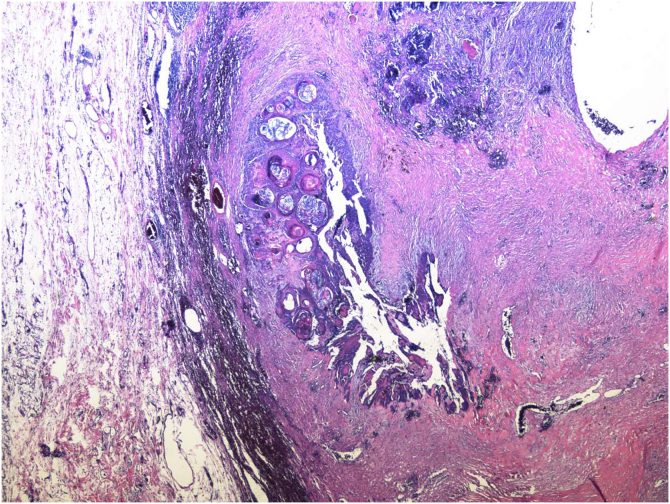
Giant pilomatricoma: well-defined proliferation, with expansive growth, adjacent to subcutaneous adipose tissue (Hematoxylin & eosin, ×200).

**Figure 3:**
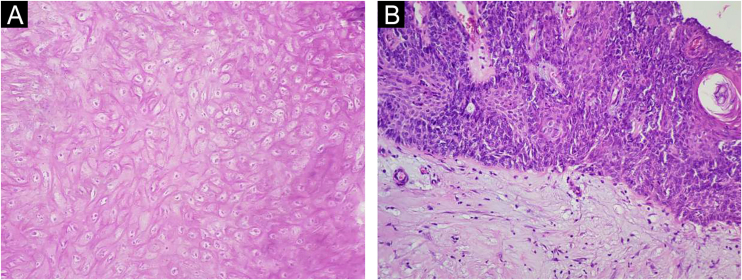
Histopathology depicting typical pilomatricoma findings such as “ghost cells” (A) and basophilic matrix cells (B) the right (Hematoxylin & eosin, ×400).

**Figure 4:**
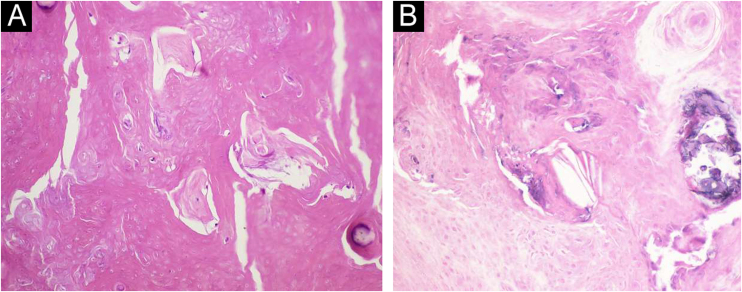
Osseous matrix foci and dystrophic calcification amid compact keratin (Hematoxylin & eosin, ×200).

Pilomatricoma or calcifying epithelioma of Malherbe with ossification is a neoplasm that is almost always benign, with fewer than 20 cases of malignant transformation described in the literature, which exhibits differentiation towards the matrix cells of the hair follicle.^1^ Clinically, it is characterized by a nodule or papule, with a normal or slightly erythematous surface, and a hard to stone-like consistency, mobile in relation to the deep planes, and slow growth,^1^, ^2^, ^3^, ^4^, ^5^ varying from 0.5 to 3cm in diameter.^2^ Most of them are solitary and asymptomatic. It predominates in female patients, with a bimodal incidence peak, from five to 15 years of age and another from 50 to 60 years of age.^1^ It is predominantly located in the head and neck, followed by the trunk and upper extremities.^1^, ^2^, ^3^, ^4^, ^5^

Histopathologically, there are three cell populations: basophilic matrix cells, similar to matrix cells of anagen phase hair follicles, acellular necrotic cells called “ghost cells”, and intermediate cells. It is also possible to find metaplastic ossification, dystrophic calcification, foreign body granulomatous reactions, and mitoses.^3^

Since its first description by Malherbe and Chenantais in 1880, several variants of this tumor have been described in the literature, such as anetodermic, lymphangiectatic, bullous, aggressive, superficial, perforating, proliferating, ossifying, cystic, pseudocystic, pigmented, acantholytic and malignant. They have a similar origin, but different clinicopathological characteristics.^4^

A pilomatricoma with exuberant osseous metaplasia can be classified as the ossifying variant. With uncertain pathogenesis, it is speculated that ossification is similar to a foreign body-type tissue reaction, specifically against the keratinous material in the “ghost cells”, aiming a physical barrier that prevents contact between the tissue affected by the tumor and healthy tissue, resembling a fibrous capsule.^4^

The perforation mechanism is common to all dermatoses with epidermal perforation, in which the pathological tissue causes irritation similar to that of a foreign body, leading to hyperplasia of the epidermis and the hair follicle epithelium. This hyperplasia will be responsible for gradually involving the tumor, pushing it to the surface to be eliminated by keratinocytes. Typically, tumor islands eliminated during pilomatricoma perforation contain calcified material.^5^

The present report describes a case of pilomatricoma in a patient outside the preferential age range and gender, classified as the ossifying and perforating variant, which is extremely rare since these are characteristics present in late-stage lesions, difficult to observe due to usual early surgical treatment.

## Financial support

None declared.

## Authors’ contributions

Vânia Olívia Coelho de Almeida: Design and planning of the study; drafting and editing of the manuscript or critical review of important intellectual content; intellectual participation in the propaedeutic and/or therapeutic conduct of the studied cases; critical review of the literature.

Ana Carolina Monteiro de Camargo: Drafting and editing of the manuscript or critical review of important intellectual content; critical review of the literature.

Meire Soares de Ataíde: Effective participation in research orientation; intellectual participation in the propaedeutic and/or therapeutic conduct of the studied cases; approval of the final version of the manuscript.

Romes José Tristão: Effective participation in research orientation; intellectual participation in the propaedeutic and/or therapeutic conduct of the studied cases; approval of the final version of the manuscript.

Tullio Novaes Silva: Collection, analysis and interpretation of data; effective participation in research orientation.

## Conflicts of interest

None declared.
